# Development and Internal Validation of a Risk Prediction Model for Falls Among Older People Using Primary Care Electronic Health Records

**DOI:** 10.1093/gerona/glab311

**Published:** 2021-10-12

**Authors:** Noman Dormosh, Martijn C Schut, Martijn W Heymans, Nathalie van der Velde, Ameen Abu-Hanna

**Affiliations:** Department of Medical Informatics, Amsterdam Public Health Research Institute, Amsterdam UMC—Location AMC, University of Amsterdam, Amsterdam, The Netherlands; Department of Medical Informatics, Amsterdam Public Health Research Institute, Amsterdam UMC—Location AMC, University of Amsterdam, Amsterdam, The Netherlands; Department of Epidemiology and Biostatistics, Amsterdam UMC—Location VU, VU University Medical Center, Amsterdam, The Netherlands; Department of Internal Medicine, Section of Geriatric Medicine, Amsterdam Public Health Research Institute, Amsterdam UMC—Location AMC, University of Amsterdam, Amsterdam, The Netherlands; Department of Medical Informatics, Amsterdam Public Health Research Institute, Amsterdam UMC—Location AMC, University of Amsterdam, Amsterdam, The Netherlands

**Keywords:** Accidental falls, Fall prediction, Fall prevention, Free text, Routinely collected data

## Abstract

**Background:**

Currently used prediction tools have limited ability to identify community-dwelling older people at high risk for falls. Prediction models utilizing electronic health records (EHRs) provide opportunities but up to now showed limited clinical value as risk stratification tool, because of among others the underestimation of falls prevalence. The aim of this study was to develop a fall prediction model for community-dwelling older people using a combination of structured data and free text of primary care EHRs and to internally validate its predictive performance.

**Methods:**

We used EHR data of individuals aged 65 or older. Age, sex, history of falls, medications, and medical conditions were included as potential predictors. Falls were ascertained from the free text. We employed the Bootstrap-enhanced penalized logistic regression with the least absolute shrinkage and selection operator to develop the prediction model. We used 10-fold cross-validation to internally validate the prediction strategy. Model performance was assessed in terms of discrimination and calibration.

**Results:**

Data of 36 470 eligible participants were extracted from the data set. The number of participants who fell at least once was 4 778 (13.1%). The final prediction model included age, sex, history of falls, 2 medications, and 5 medical conditions. The model had a median area under the receiver operating curve of 0.705 (interquartile range 0.700–0.714).

**Conclusions:**

Our prediction model to identify older people at high risk for falls achieved fair discrimination and had reasonable calibration. It can be applied in clinical practice as it relies on routinely collected variables and does not require mobility assessment tests.

Falls among community-dwelling older people are common and represent a major health problem in terms of morbidity and mortality. Around 30% of community-dwelling people aged 65 years or older fall at least once per year (1,2). Falls may result in injuries, reduced quality of life, loss of function in activities of daily living, reduced independence, and increase the risk of premature death (3,4).

Several falls prevention programs have been proven to be effective in reducing falls and fall-related injuries in community-dwelling older people (5,6). A key step in fall prevention is the identification of older people at increased risk of falling in order to facilitate effective targeting of fall preventive interventions. Many national institutes adopted the guidelines for fall prevention provided by the American Geriatrics Society (AGS) and British Geriatrics Society (BGS) (7). The AGS/BGS guideline (last update 2011) has 3 components: screening of fall-prone individuals, multifactorial fall-risk assessment, and targeted interventions. Although the risk stratification in the AGS/BGS guideline is based on expert opinion and includes 2 strong risk predictors for future falls, it has a limited predictive value to identify community-dwelling older people at higher fall risk (8,9).

For community-dwelling older people, the most widely used fall screening tool is the Timed Up and Go (TUG) test (10), and it has been recommended by AGS/BGS to assess balance and gait. Despite the fact that the TUG test is a simple test, easy to apply, and hence, suitable in a primary care setting, previous studies showed limited ability and generalizability of the TUG test to predict falls in community-dwelling older people (11,12). This inadequacy of the TUG test to identify fall-prone older adults might be attributed to the multifactorial nature of falls (11). Prediction models that better capture the multifactorial nature of falls by incorporating multiple potential risk factors may perform better in community-dwelling older people and thus serve better to identify fall-prone older individuals who would benefit from a multifactorial assessment and accompanying interventions.

The adoption of the electronic patient record (EHR) has increased during the last decade. Typically, risk factors for falls are frequently explicitly documented in the EHR such as chronic conditions and medication use. The information contained in EHR data can thus provide more information about individual risk factors for falls beyond the traditional fall-risk assessment tools. A number of studies have attempted to develop prediction models to predict future falls by the utilization of EHR data (13–16). These prediction models relied primarily on a coding system, such as the International Classification of Diseases codes, to define falls. However, falls have been found to be undercoded in EHR and administrative databases (17) and usually used for billing purposes, which in turn could result in both the omission of important predictors and limited predictive performance. An alternative source of fall-related information may be found in clinical text notes which can be leveraged to capture fall incidents (18,19). Another limitation in the studies of Ye et al. and Smith et al. (13,14) is that they combined EHR data for falls in primary care settings or ambulatory settings with an in-hospital setting. Hospital EHR comprises different older population characteristics, risk factors, and fall properties and may not represent older people in primary care settings. Our study pertains to settings in which primary care is provided by general practitioners in primary care, as in the Netherlands. Note, however, that this setting may in principle differ from ones in which older persons may receive their primary care also in outpatient clinics at hospitals.

For these reasons, we sought to address the abovementioned limitations by using EHR data entirely collected in primary care settings and by the ascertainment of falls described in free text. The aim of this study was to develop a fall prediction model for community-dwelling older people using primary care EHR data and to internally validate its predictive performance.

## Method

### Study Design, Source of Data, and Study Population

This is a retrospective population-based cohort study using routinely collected data from de-identified primary care EHR. The data were collected from 50 general practices across 5 municipalities in the province of North Holland in the Netherlands. The database originates from a data registry called the Academic General Practitioner’s Network at Academic Medical Center (AHA). This registry contains the EHRs of all general practitioners participating in the network. It contains demographic data, physiological and clinical data, diagnoses, medication use, and free text notes (in Dutch), associated with patients between 2012 and 2019.

Our study cohort includes all patients registered with any general practitioner (GP) in the network at any time in the period from 2018 to 2019. We have set the index date for the entire cohort at December 31, 2018 with a 12-month observation period before the index date for obtaining the predictors and a 12-month follow-up period after the index date to determine the occurrence of falls.

Patients were included in the study cohort if they were 65 years or older at the beginning of the observation period. The data used in this study were part of an anonymized database of routinely collected data and therefore approval of an Ethics Review Board was not necessary, and the study conformed to the Declaration of Helsinki principles.

### Outcome

The outcome was any fall during the 1-year follow-up period. Data on falls were obtained from the free text written during the follow-up period. Records that included terms related or potentially related to falls (eg, fall, fallen, and stumbled) were searched by regular expressions (Supplementary Appendix A). Because the meaning of the terms depends on the context of the sentence, each fetched record was manually inspected and annotated (by N.D.), for the presence or absence of a fall. In case of doubt, the other authors (A.A.-H., N.v.d.V., and M.C.S.) were consulted. For each patient, we marked with a binary outcome variable (0 or 1) whether a patient fell (1) or not (0) during the follow-up period.

### Candidate Predictors

Initial inspection of the data set for potential predictors was conducted based on the literature and expert knowledge. A total of 79 predictors known to be associated with falls were included. Two demographic predictors included age in years, at the beginning of the observational period, and sex. Medication predictors used during the observation period were coded using the Anatomical Therapeutic Chemical (ATC) classification system. These medications were grouped into 33 fall-risk-increasing drugs (FRID) categories (20–22) based on the ATC code (Supplementary Table 1). A binary variable was introduced for each FRID category. Each category was set to 1 for patients who received any medication from the respective category during the observation period, and 0 otherwise. The International Classification of Primary Care (ICPC) (23) is the standard for coding and classification of complaints, symptoms, and disorders in general practice in the Netherlands. ICPC codes assigned to each patient during the observation period were grouped into 43 chronic condition groups (Supplementary Table 2) according to previous classification (24,25) and expert knowledge. Each of these predictor groups was set to 1 if at least one diagnosis linked to the respective group was encountered during the observation period. We obtained the chronic conditions from previous years for patients who did not consult a GP during the observation period. Because history of falls is an important predictor of future falls (26), we incorporated it as a predictor. History of falls was defined as a fall that occurred 1 year before the follow-up period. We automated our search strategy (described above) as an algorithm for the (deterministic) identification of the value of “history of falls” (yes or no) from the free text and applied it to the observation period before the index date. This means that “history of falls” in the observation period is a (derived) candidate predictor just like the other predictors. The algorithm consists of a regular expression search for trigger words, detection of negation, and coexistence of words that either refer to traffic accidents (which are outside our definition of “fall”) or indicate that “fall” was not used in the sense of “falling” (eg, fall season). We validated the algorithm’s performance to determine history of falls in the observation period in terms of accuracy, positive predictive value (PPV), sensitivity, and specificity after manual inspection of 400 randomly selected patients.

### Missing Data and Sensitivity Analysis

We recognized 2 sources of missing values. The first was medical conditions that were not registered during the daily routine observation period but obtained from previous years (as explained above). These values were probably missing because they were not properly registered in the database during the daily routine care, although they exist in the previous years. We assumed that this type of missingness is missing completely at random because there is no specific reason why the data were not registered. The second was missing data on falls during the 1-year follow-up period because some individuals did not consult a GP, and hence the GP did not register if they were fallen and these falls could not be ascertained in the text. This missingness is most likely to be a mixture of missing at random and missing not at random because falling could be a reason to visit a GP. We conducted a sensitivity analysis to compare the predictive performance of separate models developed with and without including these individuals.

### Statistical Analysis

We adhered to the Transparent Reporting of a multivariable prediction model for Individual Prognosis Or Diagnosis (TRIPOD) guidelines (27). Logistic regression analysis was used to develop the model with fall as the outcome, and age, sex, history of fall, FRID, and chronic conditions groups as candidate predictors (variables). We accounted for patients who died in the follow-up period without experiencing a fall by giving their observation a weight of less than 1, reflecting the proportion they were observed within the follow-up year, in the analysis. To control for model complexity in order to avoid overfitting, we used the Bootstrap-enhanced penalized logistic regression with the least absolute shrinkage and selection operator penalty (Bolasso) as described in the study of Bach (28). Bolasso is a bootstrap approach of the least absolute shrinkage and selection operator (Lasso) (29), where the bootstrap resampling technique is combined with the variable selection property of Lasso, to obtain consistent variable selection. The key to Bolasso is to perturb the data by sampling *b* bootstrap samples (with replacement) and apply Lasso on every sample to allow for variable selection. Consistent variables are those that appear frequently in the resulting selected variable sets. We considered 100 bootstrap samples (*b* = 100) with a size similar to the original data set. Variables retained in all the samples were used to construct the final model using unregularized logistic regression. In order to assess the robustness of the model, we compared the predictive performance of the final model with another one developed using predictors selected in 80% of the bootstrap samples.

The performance of the model was systematically assessed using the following performance measures. Discrimination was assessed using the area under the receiver operating characteristic curve (ROCAUC), where 0.5 indicates no discrimination and 1 indicates perfect discrimination. Calibration was visually assessed using a calibration plot with loess smoothing to depict the association (30) and also by plotting the mean predicted probability against the mean observed probability for each decile as specified in the TRIPOD statement (27). Calibration refers to the degree of agreement between the predicted probabilities and the observed outcomes. We also assessed the area under precision–recall curve (PRAUC) that reflects the balance between the precision (PPV) and recall (sensitivity). Furthermore, the accuracy of the probabilistic predictions was assessed using the Brier score. Finally, we used the threshold based on the Youden index to calculate the PPV, sensitivity, and specificity.

We applied 10-fold cross-validation to internally validate the model. The entire model development, including the variable selection procedure, was repeated on each of the 10-fold of the training set and tested on the held-out fold. We calculated the median and the interquartile range (IQR) of each performance measure over 10-fold.

Data were analyzed using the R statistical software environment version 4.0 (R Foundation for Statistical Computing, Vienna, Austria), and we used the glmnet R package to perform Lasso (31). The R code used to implement Bolasso is given in Supplementary Appendix B.

## Results

### Study Population

Table 1 summarizes the main characteristics of the study population. In total, data of 36 470 eligible participants were extracted from the data set. During the 1-year follow-up period, 771 died, of which 227 experienced a fall before death. The number of participants who fell at least once was 4 778 (13.1%). A Mann–Whitney test indicated that the median age was significantly higher for fallers (76.6 years, IQR 70.7–83.3 years) than nonfallers (71.4 years, IQR 68.00–77.1 years), *p* ≤ .001. Chronic conditions were obtained from previous years for 886 (2.4%) participants, among which 41 (4.6%) fell. The number of individuals who did not consult a GP in the follow-up period was 1 389 (3.8%). History of falls was observed in 4 751 (13%) of the population. The accuracy, PPV, sensitivity, and specificity of the algorithm for the identification of history of falls in the text were 97.7%, 97.9%, 97.5%, and 97.9%, respectively. A complete list of the baseline characteristics at the observation period of the participants is described in Supplementary Table 3.

**Table 1.** Summarized Baseline Characteristics of the Study Population

Predictor	Nonfallers (*n* = 31 692)	Fallers (*n* = 4 778)
Age	71.4 [68.0–77.1]	76.6 [70.7–83.3]
Female sex	16 372 (51.7)	3 026 (63.3)
History of falls	3 385 (10.7)	1 366 (28.6)
Number of cardiovascular drugs		
None	11 509 (36.3)	1 270 (26.6)
One	5 563 (17.6)	816 (17.1)
Two	5 315 (16.8)	852 (17.8)
Three	4 316 (13.6)	764 (16.0)
Four	2 619 (8.3)	540 (11.3)
Five or more	2 370 (7.5)	536 (11.2)
Antihyperglycemic drugs	5 404 (17.1)	1 045 (21.9)
Antidepressant drugs	2 201 (6.9)	577 (12.1)
Antiepileptic drugs	1 099 (3.5)	298 (6.2)
Antiparkinson drugs	387 (1.2)	128 (2.7)
Proton pump inhibitors	12 045 (38.0)	2 533 (53.0)
Urinary incontinence drugs	785 (2.5)	236 (4.9)
Nonsteroidal anti-inflammatory drugs	4 320 (13.6)	748 (15.7)
Opioids	3 883 (12.3)	1 035 (21.7)
Anxiety disorder	899 (2.8)	205 (4.3)
Dementia	785 (2.5)	282 (5.9)
Depression	867 (2.7)	277 (5.8)
Epilepsy	287 (0.9)	79 (1.7)
Parkinson disease	298 (0.9)	103 (2.2)
Memory and concentration problems	1 959 (6.2)	667 (14.0)
Vertigo or dizziness	1 101 (3.5)	345 (7.2)
Circulatory hypertension	16 061 (50.7)	2 713 (56.8)
Cardiac arrhythmia	5 556 (17.5)	1 194 (25.0)
Coronary heart disease	4 559 (14.4)	913 (19.1)
Heart failure	1 413 (4.5)	449 (9.4)
Orthostatic hypotension	164 (0.5)	62 (1.3)
Stroke including transient ischemic attack	1 803 (5.7)	484 (10.1)
Diabetes	6 869 (21.7)	1 314 (27.5)
Kidney disease	1 072 (3.4)	198 (4.1)
Hearing disorder	4 132 (13.0)	925 (19.4)
Visual disorder	8 975 (28.3)	1 839 (38.5)
Previous injury	2 416 (7.6)	853 (17.9)
Back or neck disorder	2 872 (9.1)	638 (13.4)
Osteoarthritis	10 092 (31.8)	2 031 (42.5)
Osteoporosis	1 391 (4.4)	385 (8.1)
Rheumatoid arthritis	666 (2.1)	155 (3.2)
Vitamin deficiency	936 (3.0)	243 (5.1)
Fatigue or weakness	1 520 (4.8)	463 (9.7)
Urinary incontinence	1 553 (4.9)	537 (11.2)

*Note:* Data are presented as *n* (%) or median [IQR].

### Model Development and Specification

The Bolasso approach resulted in the inclusion of 10 predictors in the final model as given in Table 2. All the retained predictors were positively associated with future falls. History of falls in the previous year was the strongest predictor (odds ratio [OR] 2.05, 95% confidence interval [CI] 1.88–2.23), followed by depression disorder (OR 1.71, 95% CI 1.47–1.98) and problems with memory or concentration (OR 1.51, 95% CI 1.36–1.67). Increased age and female sex were associated with falls (OR 1.06, 95% CI 1.06–1.06) and (OR 1.30, 95% CI 1.21–1.39), respectively. Two medications were found to be predictors of falls, namely, proton pump inhibitors (OR 1.34, 95% CI 1.25–1.43) and opioids (OR 1.27, 95% CI 1.16–1.38). Injuries in the previous year (OR 1.42, 95% CI 1.28–1.56), osteoarthritis (OR 1.22, 95% CI 1.14–1.30), and urinary incontinence (OR 1.44, 95% CI 1.28–1.61) were associated with falls. The predicted probability can be calculated using the formula $\frac{1}{1+e^{-LP}}$, where LP (linear predictor) is equal to −6.92 + 0.06 × age + 0.26 × female sex + 0.72 × history of falls + 0.29 × use of proton pump inhibitors + 0.24 × use of opioids + 0.35 × previous injury + 0.54 × depression + 0.20 × osteoarthritis + 0.36 × urinary incontinence + 0.41 × memory and concentration problems.

**Table 2.** The Final Prediction Model for Future Falls in Community-Dwelling Older Adults as Derived From the GPs Data

Predictor	Coefficient	OR (95% CI)*
Intercept	−6.92*	
Age†	0.06	1.06 (1.06–1.06)
Female sex	0.26	1.30 (1.21–1.39)
History of falls	0.72	2.05 (1.88–2.23)
Use of proton pump inhibitors	0.29	1.34 (1.25–1.43)
Use of opioids	0.24	1.27 (1.16–1.38)
Previous injury	0.35	1.42 (1.28–1.56)
Depression	0.54	1.71 (1.47–1.98)
Osteoarthritis	0.20	1.22 (1.14–1.30)
Urinary incontinence	0.36	1.44 (1.28–1.61)
Memory and concentration problems	0.41	1.51 (1.36–1.67)

*Notes:* OR = odds ratio; CI = confidence interval; GP = general practitioner. The numbers are rounded to 2 decimal places.

*The 95% CI of the intercept’s coefficient is −7.25 to −6.60.

^†^The OR of the age is based on each year increase.

*All predictors reached *p* < .001.

### Model Performance and Validation

Table 3 presents the model’s predictive performance after performing 10-fold cross-validation. The ability of the model to discriminate between fallers and nonfallers measured by the ROCAUC had a median of 0.705 (IQR 0.700–0.714). The median PRAUC was 0.290 (IQR 0.278–0.298). The median PPV was 0.238 (IQR 0.223–0.256). The median Brier score was 0.105 (IQR 0.103–0.108).

**Table 3.** The Predictive Performance of the Final Prediction Model Based on 10-Fold Cross-Validation

Measure	Median	Interquartile Range
ROCAUC	0.705	0.700–0.714
PRAUC	0.290	0.278–0.298
Sensitivity	0.623	0.593–0.664
Specificity	0.698	0.665–0.740
PPV	0.238	0.223–0.256
Brier score	0.105	0.103–0.108

*Notes:* ROCAUC = area under the receiver operating characteristic curve; PRAUC = area under precision–recall curve; PPV = positive predictive value. The numbers are rounded to 3 decimal places.

**Figure 1.**
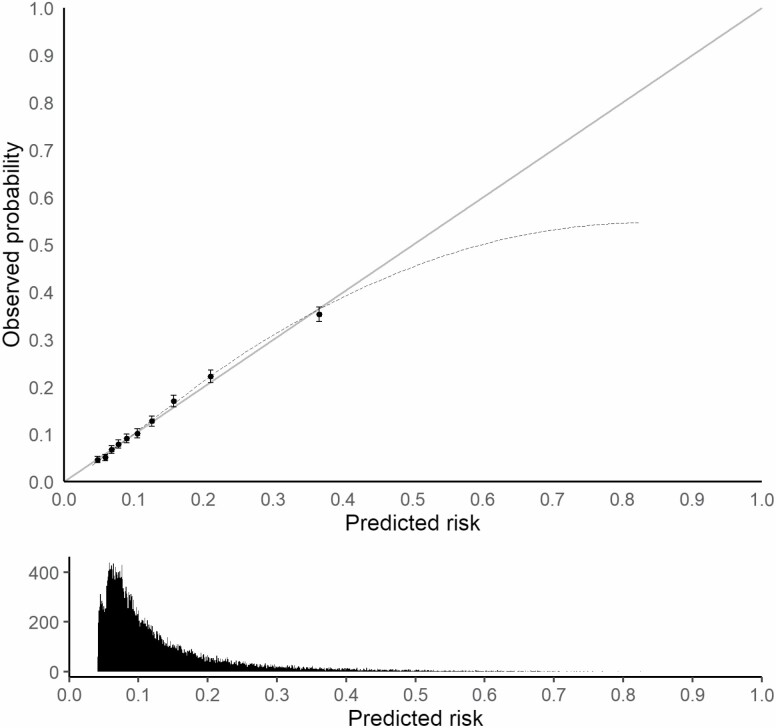
The calibration plot of the final falls prediction model. The calibration plot demonstrates the relation between the predicted and observed falls rate. The diagonal line represents the performance of an ideal model. The dashed line represents the actual model performance that compares the predicted and observed falls probabilities (using 10-fold cross-validation). Points estimated below the diagonal line reflect over prediction, whereas points located above the diagonal line reflect under prediction. The graph in the lower compartment of the figure shows a histogram of the distribution of the predicted falls probabilities.

Figure 1 depicts the calibration plot of the model. The diagonal line represents the performance of an ideal model. Points estimated below the diagonal line reflect overprediction, whereas points located above the diagonal line reflect underprediction.

The number of predictors that appeared in 80% of the bootstrap samples or more was 21 (Supplementary Table 4). After fitting a model with all these predictors, the median ROCAUC was 0.716 (IQR 0.712–0.721); PRAUC 0.293 (IQR 0.293–0.304); sensitivity 0.690 (IQR 0.630–0.719); specificity 0.673 (IQR 0.622–0.700); PPV 0.235 (IQR 0.218–0.24); and the Brier score 0.104 (0.102–0.108).

### Sensitivity Analysis

Supplementary Table 5 illustrates the predictive performance of 2 models developed after the exclusion of individuals whose predictors of the chronic condition groups were obtained from previous years (Model 1) and individuals who did not consult a GP in the follow-up period (Model 2). Supplementary Figure 1 shows the calibration plots of these 2 models. Both models demonstrated similar discrimination and calibration compared to the final prediction model.

## Discussion

In this study, we have developed and internally validated a prediction model for falls in community-dwelling older people using structured and free-text data of a large primary care EHR data collected from a network of general practitioners. The model displayed fair discrimination and reasonable calibration at low values and consistent overprediction at high values of predicted risk. The overprediction, however, pertains to a relatively small group of patients. The final prediction model included age, sex, history of falls, 2 medications, and 5 medical conditions.

The results of this study confirm findings of earlier studies that identify history of falls as the strongest predictor for future falls, with age and female sex playing an important role in the prediction. In addition, our model showed that 5 medical conditions and 2 medications were also associated with falls. The assessment of these risk factors provides better fall-risk estimation and higher-predictive performance beyond the screening algorithm of the AGS/BGS guideline and the TUG test, to identify fall-prone community-dwelling older people. The TUG test could be used to assess simple balance and mobility function but it may not be sufficiently broad to address other fall-risk components, including, among others, chronic conditions and certain medications. Furthermore, our model can be easily applied in clinical practice as it contains variables routinely collected and readily available in the EHR and does not require additional mobility assessment tests.

The discriminative ability of our model was fair and comparable to previously published models incorporating EHR data partially collected in primary care settings (15,16), models developed using insurance claims (32), and models based on research cohorts (33–35). Our study cohort included a large multicenter sample of community-dwelling older people whose data were extracted from an EHR entirely collected in the primary care setting, unlike prediction models developed using a combination of GP data and hospital data (15), data collected in an ambulatory setting (16), and data extracted from insurance claims (32). Therefore, our study sample is more likely to represent community-dwelling older people, and falls were more likely to have been occurring in the community (not inpatient falls). Moreover, the model was internally validated by means of cross-validation, and a sensitivity analysis was conducted to avoid bias. This is in contrast to previously developed models of studies (15,34,36), which were not validated and the models of studies (16,32) where only a single random split (1 for model development and 1 for internal validation) was used, which has been shown to be inadequate for validation (37). Only the model of Palumbo et al. (35) was internally validated using cross-validation.

The prevalence of falls among community-dwelling older people in this study was higher, compared to previous studies conducted using EHR data with falls as outcome (13,16) and falls combined with fractures (14,15). This can be explained by the difference in fall ascertainment. We relied on free-text notes where information on falls is naturally documented to determine fallers, while the abovementioned studies used classification codes that are subject to inaccurate documentation by GPs or the administration staff of the practice. Our approach is in line with 2 other studies (18,19) that also recognized the advantage of using clinical free text to identify falls and fall-related injuries.

Our final model included 10 predictors. The fact that these predictors were a combination of demographic, chronic medical conditions, and medications is concordant with the multifactorial nature of falls. History of falls, increased age, female sex, and the presence of certain medical conditions, namely, depression, urinary incontinence, and osteoarthritis, were previously reported as independent risk factors for falls (26,38). Among the medications, the use of opioids and proton pump inhibitors was also recognized to be positively associated with falling risk in multiple meta-analyses studies (22,39,40). On the other hand, psychotropic agents, which are the most commonly offending group of medications associated with an increased risk of falls (21), were not found to be predictive in our results. This may have been caused by the low prevalence of use in older—mostly community-dwelling—primary care patients. It must be emphasized that, because we developed a prediction and not a causal model, the inclusion of other significantly correlated predictors with falls does not necessarily lead to improvements in prediction.

There are some similarities between the predictors retained in our prediction model and those described in other prediction models developed using community-based research cohorts. Our results are consistent with those of Bongue et al. (33,34,41,42) who found that history of falls and female sex are important predictors for falls. In accordance with the finding of Bongue et al. (33), we found that the presence of osteoarthritis is a predictor for falls. However, in contrary to our prediction model, depression was not retained in their final model. One possible explanation for this is the presence of psychoactive medications, which could serve as a proxy for depression. The authors also showed that the presence of urinary incontinence was not predictive, contrary to our results that also corroborate the findings of Tromp et al. (34). These results are likely to be related to the difference in patient age between our study and the one of Tromp et al. (34), on the one hand, and the study of Bongue et al. (33), on the other hand.

Recall, that although our prediction model could be useful to identify community-dwelling older people at higher fall risk, the association of the predictors and falls does not imply causality and, therefore, should be interpreted with caution. For example, proton pump inhibitors are commonly used to treat acid-related gastrointestinal diseases or to protect the stomach of polypharmacy patients (43). Proton pump inhibitors therapy has also been associated with functional decline and with fracture risk that might increase fall risk (22,40). However, there is currently no evidence that shows a causal relationship between the use of proton pump inhibitors and falls. The existence of this predictor in the prediction model may be a surrogate of an underlying disease or an indication of frailty in older people who often have multiple chronic conditions requiring multiple medications.

With respect to the sensitivity analysis, the exclusion of individuals whose predictors of the chronic condition groups were obtained from previous years and individuals who did not consult a GP in the follow-up period did not affect the predictive performance as indicated in the overlapping IQR values of the performance measures. This is an indication that missing data did not affect the models generated and that missing values were largely unrelated to reasons related to falling. In addition, our strategy to retain predictors that appeared in all bootstrap samples is robust in terms of the obtained predictive performance and the parsimony of the model. The ROCAUC was slightly improved when using the predictors retained from 80% of the bootstrap samples. However, this simple improvement is accompanied by the inclusion of 11 more predictors (21 predictors in total) which may compromise the usability of the model. This finding was also reported by Palumbo et al. (35) who found that the ROCAUC could be improved by including more predictors until plateau level is reached.

Another important aspect to consider that is rarely assessed in falls prediction models (44) is the assessment of calibration performance. The current study found that the predicted probabilities of fall and the observed probabilities agreed over almost the whole range of probabilities. Only when the predicted probability is more than 0.45, then the prediction overestimates the proportion of observed fallers. That means that, alike to existing models and tools, clinicians should be aware that our model overestimates falling risk for individuals at higher risk of falling, which in turn might lead to unnecessary interventions. However, these individuals constituted less than 1% of the study sample, and clinicians are inclined to overtreat in case of falls prevention as the benefits of the interventions generally outweigh the harms. The overprediction pertains to a relatively small group of patients. We have refitted the model when including an extra covariate representing the number of comorbidities, in order to inspect whether calibration might improve. However, this was not the case (model and graph are not shown).

The findings of this study have a number of implications. The predictors identified in our prediction model are variables readily accessible in the EHR and routinely obtained in a primary care setting. For clinical practice, the integration of this fall-risk stratification model in an EHR allows the clinicians to identify high-risk individuals in order to offer them interventions adapted to their needs. Furthermore, our approach of using Bolasso for variable selection was generally robust offering a balance between performance and interpretability. Researchers can use this technique to simplify prediction models for complex problems when a large number of variables are considered. Finally, our study highlights the importance of introducing a specific code for falls in the ICPC coding system in the future. While there are many codes for injuries (eg, A80 Trauma/Injury) that may result from falls, noninjurious falls are much more common and usually precede injuries. There is, therefore, a definite need for a code to describe falls to facilitate data retrieval, aggregation, and the development of prediction models.

A limitation of this study is the exclusion of laboratory measurements (eg, blood pressure, blood glucose) from the analysis. We chose to discard these predictors because of the large number of individuals who did not have these measured (range 46%–100%). We attempted to predict falls using imputed values of the missing laboratory measurements but that did not improve the predictive performance. We also tried the extreme gradient boosting (XGBoost) (45) machine learning algorithm to predict falls by including the missing laboratory measurements. XGBoost is a gradient tree boosting-based method with extensions. One of the interesting extensions is the sparsity awareness that can handle the existence of missing values. Nevertheless, our experiments with the use of XGBoost revealed that these variables were not predictive. Another limitation is that our study underestimates the prevalence of falls as not all falls are reported to or documented by the GPs. Older people tend to not report falls, or even forget, unless medical attention is required (46). In addition, our data did not contain predictors on other important fall-risk factors such as mobility, gait, or environmental factors. Nevertheless, some of the other predictors (eg, osteoarthritis, previous injury) may have been a proxy for functional limitations. Furthermore, our results rely on accurate documentation of the GPs and the use of appropriate codes during a consultation visit or information gathered from other caregivers. Some information may not always get recorded, such as medications prescribed by specialists after a hospital visit, and hence, some risk factors might be underestimated.

While our manual search strategy to identify fallers was crucial to detect all possible falls in clinical notes, future studies could consider machine learning to detect fallers in clinical notes to build prediction models or identify fall-risk factors as, for example, deployed in the work of Womack et al. (47). When effective, these approaches could avoid laborious manual labeling. Future studies are needed to externally validate our prediction model and to test its applicability for screening in a GP setting where older individuals are at increased risk of falling and would thus benefit from a multifactorial assessment and intervention in adherence with the global falls guidelines initiative of Montero-Odasso et al. (48) for fall management and prevention. We also intend to validate this model using another independent large data set collected in another, but similar, primary care setting.

## Supplementary Material

glab311_suppl_Supplementary_Material
