# Supplementary material for: Development and Internal Validation of a Risk Prediction Model for Falls Among Older People Using Primary Care Electronic Health Records
Source: J Gerontol A Biol Sci Med Sci. 2021 Oct 12;77(7):1438–45. doi: 10.1093/gerona/glab311 (PMC9255681; doi:10.1093/gerona/glab311)
Supplement: glab311_suppl_Supplementary_Material [file glab311_suppl_supplementary_material.pdf]

Supplementary material

Online only documents content:

eTable 1. Fall-risk increasing drugs (FRID) with the corresponding ATC code used in the aggregation

eTable 2. Chronic conditions and diseases with the corresponding ICPC codes used in the aggregation

eTable 3. Baseline characteristics of the study population

eTable 4. Frequency of predictor selection after the application of Bolasso using 100 bootstrap samples

eTable 5. The results of the sensitivity analysis performed to assess the effect of missing values on the predictive performance

eFigure 1. The calibration plots of model 1 (excluding individuals with missing chronic conditions) and model 2 (excluding individuals with missing outcome)

Appendix A. Trigger words used to search for falls in free-text

Appendix B. The R code to perform Bolasso

eTable 1. Fall-risk increasing drugs (FRID) with the corresponding ATC code used in the aggregation

FRID	ATC codes
Proton pump inhibitors	A02BC
Nitrates	C01DA
Alpha and beta blocking agents	C07AG
Non-dihydropyridine CCBs	C08D
Dihydropyridine CCBs	C08C
ARBs	C09CA
ACEIs	C09AA
Non-selective beta blockers	C07AA
Selective beta blockers	C07AB
Alpha blockers	C02CA
Antiadrenergics	C02A,C02B,C02C
Antiarrhythmic	C01B
Diuretics	C03
High ceiling diuretic	C03C
Low ceiling diuretic	C03A,C03B
Cardiac glycosides	C01A
Biguanides	A10BA
Insulins and analogues	A10A
sulfonylureas	A10BB
Vasodilators used in cardiac diseases	C01D
Statins	C10AA
Urinary incontinence drugs	G04BD
Alpha blockers used in BPH	G04CA
Opioids	N02A
Antiepileptic drugs	N03
Antiparkinson drugs	N04
Non-selective MRIs	N06AA
SSRIs	N06AB
Other antidepressants	N06AX
Anxiolytics	N05B
Hypnotics and sedatives	N05C
Antipsychotics	N05A
NSAIDs	M01AA, M01AB, M01AC, M01AE,M01AG ,M01AH

Note. CCBs = Calcium Channel Blockers; ARBs = Angiotensin Receptor Blockers; ACEIs = Angiotensin Converting Enzyme Inhibitors; BPH = Benign prostatic hyperplasia; MRIs = Monoamine Reuptake Inhibitors; SSRIs = Selective Serotine Reuptake Inhibitors; NSAIDs = Non-steroidal Anti-inflammatory Drugs.

eTable 2. Chronic conditions and diseases with the corresponding ICPC codes used in the aggregation

Chronic condition/disease	ICPC codes
Anxiety	P01,P74,P79,P75
Asthma	R96
Blood disorders	B78,B80,B81,B82
Cancer	A79,B72,B73,B74,D74,D75, D77,N74,R84,R85,S77,T71, U75,U76,U77,X76,W72,X75, X77,Y77,Y78, F74.01,H75.01,K72.01,A79
Cardiac arrhythmia	K05,K80,K04,K84.01,K84.02, K84.07,K78,K79,A79
Chronic alcohol abuse	P15
Chronic back or neck disorders	L83,L84,L85,L86,L03
Chronic ulcerative colitis	D94
Chronic kidney diseases	U88,U75,U95
Chronic obstructive pulmonary disease	R91,R95
Chronic skin problems	S08,S78,S79,S81,S82,S83, S86,S87,S88,S91,S97,S99
Coronary heart disease	K74,K75,K76
Digestive Constipation	D12
Dementia	P70
Depression	P03,P76
Diabetes	T90,N94.02,F83.01,K99.06
Epilepsy	N88
Fracture	L72,L74,L76,L73,L75
Gastric or duodenal ulcer	D85,D86
Hearing disorder	H84,H85,H02,H86
Heart failure	K77
Injury	S19,S18,S17,A81,A80, S16,N80,A82,L81
Insomnia	P06
Circulatory Hypertension	K85,K86,K87
Multiple sclerosis	N86
Neurological Cephalalgia	N01,N89,N90,N02,N03,N92
Osteoarthritis	L89,L90,L91,L87,L92,L94,L98
Osteoporosis	L95
Other mental disorders	P15,P71,P72,P73,P80, P85,T06,P98,P99
Parkinson disease	N87,N87.01
Rheumatoid arthritis	L88
Schizophrenia	P72
Stroke including transient ischemic attack	K89,K90

Chronic condition/disease	ICPC codes
Allergy	A12,A92,F71,R97,S88
Thyroid disorders	T15,T71,T72,T81, T85,T86,T99.02
Dyslipidemia	T93
Urinary incontinence	U04,P12
Visual disorder	F83,F84,F92,F93,F94,F91
Vertigo and dizziness	N82,N17
Orthostatic hypotension	K88
Vitamin deficiencies	T91
Fatigue and weakness	A04
Memory and concentration problems	P20

eTable 3. Baseline characteristics of the study population

Predictor	Non-fallers (n=31,692)	Fallers (n=4,778)
Age	71.41 [67.99, 77.06]	76.57 [70.74, 83.31] *
Female sex	1,6372 (51.7)	3,026 (63.3) *
History of falls	3,385 (10.7)	1,366 (28.6) *
Proton pump inhibitors	12,045 (38.0)	2533 (53.0) *
Nitrates	2,206 (7.0)	536 (11.2) *
Alpha and beta blocking agents	210 (0.7)	40 (0.8)
Non-dihydropyridine CCBs	841 (2.7)	210 (4.4) *
Dihydropyridine CCBs	6,072 (19.2)	1,002 (21.0) *
ARBs	3,649 (11.5)	718 (15.0) *
ACEIs	6,486 (20.5)	1,080 (22.6) *
Non-selective beta blockers	755 (2.4)	169 (3.5) *
Selective beta blockers	8,123 (25.6)	1,529 (32.0) *
Alpha blockers	329 (1.0)	57 (1.2)
Antiadrenergics	346 (1.1)	59 (1.2)
Antiarrhythmics	624 (2.0)	173 (3.6) *
Diuretics	7,829 (24.7)	1,565 (32.8) *
High ceiling diuretics	2,261 (7.1)	690 (14.4) *
Low ceiling diuretics	5,353 (16.9)	831 (17.4)
Cardiac glycosides	500 (1.6)	122 (2.6) *
Biguanides	4,611 (14.5)	852 (17.8) *
Insulins and analogues	1,511 (4.8)	354 (7.4) *
Sulfonylureas	2,094 (6.6)	402 (8.4) *
Vasodilators used in cardiac diseases	2,208 (7.0)	536 (11.2) *
Statins	12,744 (40.2)	2,188 (45.8) *
Urinary incontinence drugs	785 (2.5)	236 (4.9) *
Alpha blockers used in BPH	2,005 (6.3)	331 (6.9)
Opioids	3,883 (12.3)	1,035 (21.7) *
Antiepileptic drugs	1,099 (3.5)	298 (6.2) *
Antiparkinson drugs	387 (1.2)	128 (2.7) *
Non-selective MRIs	790 (2.5)	219 (4.6) *
SSRIs	968 (3.1)	244 (5.1) *
Other antidepressants	570 (1.8)	154 (3.2) *
Anxiolytics	2,529 (8.0)	564 (11.8) *
Hypnotics and sedatives	2,417 (7.6)	595 (12.5) *
Antipsychotics	583 (1.8)	151 (3.2) *
NSAIDs	4,320 (13.6)	748 (15.7) *
Anxiety	899 (2.8)	205 (4.3) *
Asthma	2,434 (7.7)	502 (10.5) *
Blood disorders	1,240 (3.9)	323 (6.8) *

Predictor	Non-fallers (n=31,692)	Fallers (n=4,778)
Cancer	3,604 (11.4)	692 (14.5) *
Cardiac arrhythmia	5,556 (17.5)	1,194 (25.0) *
Chronic alcohol abuse	518 (1.6)	108 (2.3) *
Chronic back or neck disorder	2,872 (9.1)	638 (13.4) *
Chronic ulcerative colitis	126 (0.4)	25 (0.5)
Chronic kidney disease	1,072 (3.4)	198 (4.1) *
COPD	2,960 (9.3)	610 (12.8) *
Chronic skin problems	8,486 (26.8)	1,587 (33.2) *
Coronary heart disease	4,559 (14.4)	913 (19.1) *
Digestive constipation	1,224 (3.9)	362 (7.6) *
Dementia	785 (2.5)	282 (5.9) *
Depression	867 (2.7)	277 (5.8) *
Diabetes	6,869 (21.7)	1,314 (27.5) *
Epilepsy	287 (0.9)	79 (1.7) *
Fracture	680 (2.1)	204 (4.3) *
Gastric or duodenal ulcer	102 (0.3)	29 (0.6) *
Hearing disorder	4,132 (13.0)	925 (19.4) *
Heart failure	1,413 (4.5)	449 (9.4) *
Injury	2,416 (7.6)	853 (17.9) *
Insomnia	989 (3.1)	212 (4.4) *
Circulatory hypertension	16,061 (50.7)	2,713 (56.8) *
Multiple sclerosis	43 (0.1)	14 (0.3) *
Neurological cephalalgia	3,510 (11.1)	766 (16.0) *
Osteoarthritis	10,092 (31.8)	2,031 (42.5) *
Osteoporosis	1,391 (4.4)	385 (8.1) *
Other mental disorders	1,357 (4.3)	343 (7.2) *
Parkinson disease	298 (0.9)	103 (2.2) *
Rheumatoid arthritis	666 (2.1)	155 (3.2) *
Schizophrenia	92 (0.3)	14 (0.3)
Stroke including TIA	1,803 (5.7)	484 (10.1) *
Allergy	7,936 (25.0)	1,456 (30.5) *
Thyroid disorders	937 (3.0)	192 (4.0) *
Dyslipidemia	2,670 (8.4)	360 (7.5) *
Urinary incontinence	1,553 (4.9)	537 (11.2) *
Visual disorder	8,975 (28.3)	1,839 (38.5) *
Vertigo and dizziness	1,101 (3.5)	345 (7.2) *
Orthostatic hypotension	164 (0.5)	62 (1.3) *
Vitamin deficiency	936 (3.0)	243 (5.1) *
Fatigue and weakness	1,520 (4.8)	463 (9.7) *
Memory and concentration problems	1,959 (6.2)	667 (14.0) *

Note. Data are presented as n (%) or median [IQR]. CCBs = Calcium Channel Blockers; ARBs = Angiotensin Receptor Blockers; ACEIs = Angiotensin Converting Enzyme Inhibitors; BPH = Benign prostatic hyperplasia; MRIs = Monoamine

Reuptake Inhibitors; SSRIs = Selective Serotine Reuptake Inhibitors; NSAIDs = Non-steroidal Anti-inflammatory Drugs; COPD = Chronic Obstructive Pulmonary Disease; TIA = Transient Ischemic Attack.
*p < .05

eTable 4. Frequency of predictor selection after the application of Bolasso using 100 bootstrap samples

Predictor	Number of selection times
Age	100
Female sex	100
History of falls	100
Proton pump inhibitors	100
Opioids	100
Depression	100
Injury	100
Osteoarthritis	100
Urinary incontinence	100
Memory and concentration problems	100
Fatigue weakness	99
Vertigo dizziness	96
Stroke including transient ischemic attack	93
Diabetes	91
Neurological Cephalalgia	88
Osteoporosis	86
Other mental disorders	86
High ceiling diuretic	85
Cardiac arrhythmia	82
Visual disorder	82
Antiepileptics	80
Urinary incontinence drugs	78
Vitamin deficiency	77
Parkinson disease	74
Chronic skin problems	72
Hearing disorder	69
Hypnotics and sedatives	59
Non-selective monoamine reuptake inhibitors	58
Systemic diseases Allergy	56
Antiparkinson drugs	55
Selective serotonin reuptake inhibitors	53
Digestive Constipation	53
Antiarrhythmics	49

Predictor	Number of selection times
Insulins and analogues	36
Statins	34
Asthma	34
Epilepsy	30
Chronic obstructive pulmonary disease	29
Cancer	28
Chronic back or neck disorder	27
Heart failure	26
Biguanides	22
Non-dihydropyridine calcium channel blockers	20
Other antidepressants	20
Anxiolytics	16
Orthostatic hypotension	16
Rheumatoid arthritis	15
Dementia	14
Nitrates	13
Gastric or duodenal ulcer	13
Angiotensin receptor blocker	11
Multiple sclerosis	10
Anxiety disorders	6
Blood disorders	6
Diuretics	5
Non-selective beta blocking agents	4
Sulfonylurea	4
Chronic alcohol abuse	4
Fracture	4
Chronic kidney disease	3
Non-steroidal anti-inflammatory drugs	2
Insomnia	2
Circulatory Hypertension	2
Thyroid disorders	2
Selective beta blocking agents	1
Coronary heart disease	1
Dyslipidemia	1
Alpha and beta blocking agents	0
Dihydropyridine calcium channel blockers	0
Angiotensin converting enzyme inhibitors	0
Alpha blockers	0
Antiadrenergic	0
Low ceiling diuretic	0
Cardiac glycosides	0

Predictor	Number of selection times
Vasodilators used in cardiac diseases	0
Alpha blockers used in benign prostatic hyperplasia	0
Antipsychotics	0
Chronic ulcerative colitis	0
Schizophrenia	0

eTable 5. The results of the sensitivity analysis performed to assess the effect of missing values on the predictive performance

Measure	Model 1 [a]	Model 2 [b]
ROCAUC	0.702 [0.698-0.714]	0.711 [0.704-0.720]
PRAUC	0.290 [0.275-0.304]	0.293 [0.277-0.296]
Sensitivity	0.614 [0.591-0.672]	0.644 [0.569-0.677]
Specificity	0.700 [0.664-0.722]	0.693 [0.646-0.722]
PPV	0.243 [0.234-0.251]	0.24 [0.228-0.255]
Brier score	0.109 [0.106-0.111]	0.107 [0.107-0.109]

Note. Values are presented as median [IQR] of the 10 folds cross-validation. The numbers are rounded to three decimal places

[a] The performance of the model after excluding individuals with missing diagnoses

[b] The performance of the model after excluding individuals with missing outcomes (did not contact a GP in the follow-up period)

eFigure 1. The calibration plots of model 1 (excluding individuals with missing chronic conditions) and model 2 (excluding individuals with missing outcome)

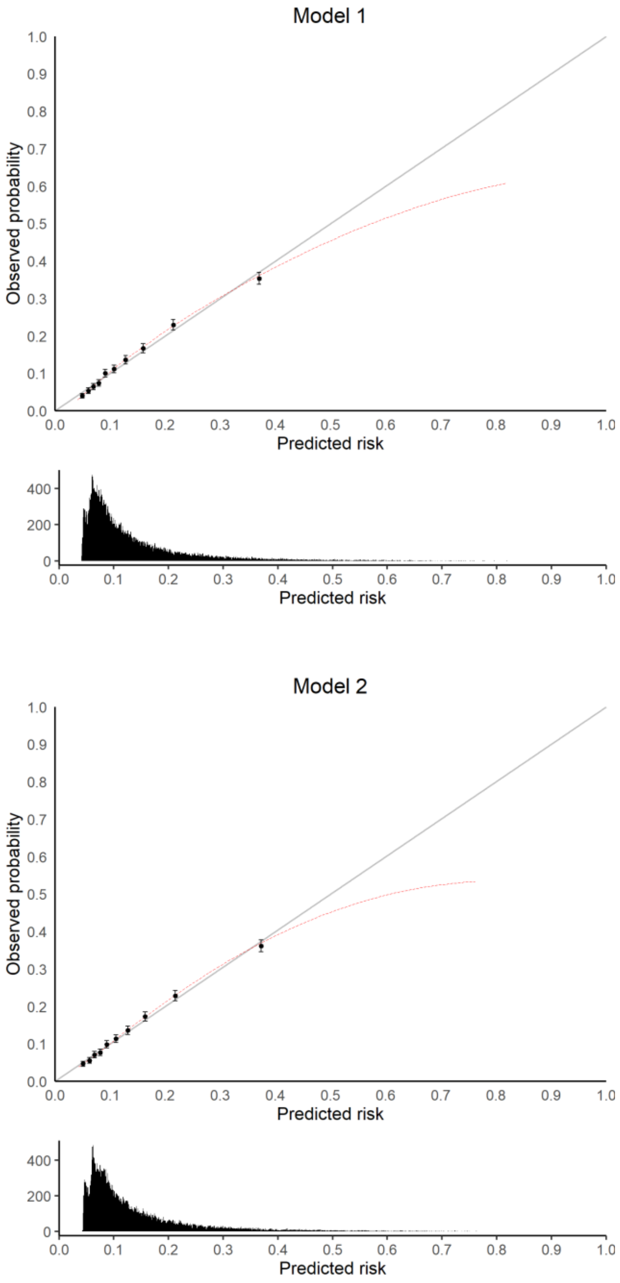

These plots demonstrate the relation between the predicted and observed falls rate. The diagonal line represents the performance of an ideal model. The dashed line represents the actual model performance that compares the predicted and observed falls probabilities (using 10 folds cross-validation). Points estimated below the diagonal line reflect over prediction, whereas points located above the diagonal line reflect under prediction. The graph in the lower compartment of each plot shows a histogram of the distribution of the predicted falls probabilities.

Appendix A. Trigger words used to search for falls in free-text

List of falling trigger words used to search the clinical free-text and the regular expression pattern. The English translation of each word is given between parentheses.

List of words:

- val (fall)
- gevallen (fallen)
- viel (fell)
- omgevallen (fallen over)
- gestruikeld (tripped)
- struikelde (tripped)
- uitgegleden (slipped)
- uitglijden (slip)
- gleed (slipped)
- flauwgevallen (fainted)

Regular expression pattern:

```
\bval\b|\bgevallen\b|\bviel\b|\bomgevallen\b|gestruikeld|struikelde|uitgegleden|uitglijden|gleed|flauwgevallen
```

Appendix B. The R code to perform Bolasso

```
library(glmnet)

set.seed(1506)
df = data.frame(outcome=sample(c(0,1), replace=TRUE, size=100),
                var1=rnorm(100, mean=10, sd=2),
                var2=rnorm(100, mean=2, sd=1))

y = df$outcome
x = sparse.model.matrix(~.-1, df[, !colnames(df) %in% c("outcome") ])

predictor_names = colnames(x)
coef_grid = matrix(nrow=length(predictor_names)+1, ncol = 0)

rank = 100
B = 100 # number of bootstrap samples
Nfolds = 10 # number of folds in CV

for (b in 1:B)
{
  cat(b, fill=T)

  df_boot = cbind(outcome=y,x)
  df_boot = df_boot[sample(rownames(df_boot),size = nrow(df_boot),
replace = T),]

  y_boot = df_boot[,1]
  x_boot = df_boot[,!colnames(df_boot) %in% c("outcome")]

  glmnet_fit = cv.glmnet(x=x_boot, y=y_boot, alpha=1, family='binomial',
nfolds = nfolds)
  my_coef <- coef(glmnet_fit, s="lambda.1se")
  coef_grid = cbind(coef_grid, my_coef[,1])
}


selected_predictors = coef_grid[rowSums(coef_grid!=0)>=rank, ]
selected_predictors = rownames(selected_predictors)
```
